# Retraction: Ectopic Expression of a Maize Hybrid Down-Regulated Gene *ZmARF25* Decreases Organ Size by Affecting Cellular Proliferation in *Arabidopsis*

**DOI:** 10.1371/journal.pone.0155904

**Published:** 2016-05-12

**Authors:** The PLOS ONE Editors

It has been brought to the attention of the *PLOS ONE* Editors that there is partial duplication of images in Figures 4B, 4C and 5A.

Upon our follow up with the authors of the article, it was established that images for sample 35S::*ZmARF25* plant #4 panel 4B (siliques) and 4C (plants) were generated by Photoshop software manipulation of images from other plants. In Figure 5A, the same image was erroneously used for both samples.

In view of the intentional manipulation of photographic images related to sample 35S::*ZmARF25* plant #4, the *PLOS ONE* Editors retract this article. The authors expressed deep regret for this circumstance and apologize to the community.
